# Scene Buildup From Latent Memory Representations Across Eye Movements

**DOI:** 10.3389/fpsyg.2018.02701

**Published:** 2019-01-11

**Authors:** Andrey R. Nikolaev, Cees van Leeuwen

**Affiliations:** Laboratory for Perceptual Dynamics, Brain & Cognition Research Unit, KU Leuven, Leuven, Belgium

**Keywords:** eye movement, visual scene, brain activity, latent representations, working memory

## Abstract

An unresolved problem in eye movement research is how a representation is constructed on-line from several consecutive fixations of a scene. Such a scene representation is generally understood to be sparse; yet, for meeting behavioral goals a certain level of detail is needed. We propose that this is achieved through the buildup of latent representations acquired at fixation. Latent representations are retained in an activity-silent manner, require minimal energy expenditure for their maintenance, and thus allow a larger storage capacity than traditional, activation based, visual working memory. The latent representations accumulate and interact in working memory to form to the scene representation. The result is rich in detail while sparse in the sense that it is restricted to the task-relevant aspects of the scene sampled through fixations. Relevant information can quickly and flexibly be retrieved by dynamical attentional prioritization. Latent representations are observable as transient functional connectivity patterns, which emerge due to short-term changes in synaptic weights. We discuss how observing latent representations could benefit from recent methodological developments in EEG-eye movement co-registration.

## Scene Buildup Across Eye Movements

From finding our keys in a room to appreciating a work of art, various aims for visually exploring a scene seem to require a rich, integral representation. Task-relevant details acquired during fixation would accumulate therein, across multiple eye movements, on a time scale of, roughly, one to a few minutes (Melcher and Kowler, 2001; Hollingworth and Henderson, 2002; Tatler et al., 2003; Melcher, 2006).

However, it remains a matter of debate how much of the information obtained at fixation is, in fact, memorized across saccades. Some authors have argued that such memorization is not needed at all, as the world itself serves as an “outside memory” (O’Regan and Noë, 2001). Others claim that memory is used only to a limited extent; object information is likely to be preserved between saccades, but only as long as it remains in the focus of attention (Rensink, 2000). The majority of researchers assign memory a somewhat larger role, and propose that representations of several fixated objects are accumulated in visual short term memory (VSTM) (Irwin, 1996; Irwin and Zelinsky, 2002; Prime et al., 2011; Tatler and Land, 2011; Higgins and Rayner, 2015).

Visual short term memory may function as “*trans*-saccadic memory” to provide visual stability across saccades. This function could be achieved through remapping between pre- and post-saccadic visual representations (Mathôt and Theeuwes, 2011; Marino and Mazer, 2016). However, VSTM capacity limitations restrict the information accumulating to 3–5 items, and when this number is exceeded, newly incoming information will overwrite the old. These limitations would typically keep the resulting scene representation sparse (Irwin, 1996; Irwin and Zelinsky, 2002). The presumed sparseness clashes with average observers’ ability to successfully recognize thousands of scene images (Standing, 1973; Konkle et al., 2010). Large amounts of information, moreover, can be retained about both a scene’s spatial layout and the objects therein (Friedman, 1979; Sanocki et al., 2010). These findings suggest a special aptness of memory for scenes may exist.

Scene representation across eye movement, according to the ongoing, task-related character of information processing could be a function of “working memory” (Baddeley, 1992; Melcher, 2006). Besides information in the focus of attention, for which capacity is limited, working memory also encompasses an activated portion of visual long-term memory (VLTM) (Cowan, 1988; Oberauer, 2002). The latter may have a role in maintaining information in a heightened state of availability, in particular information about recently attended items or information associated with items in the attentional focus. The involvement of VLTM would allow detailed information from many locations to be used for constructing a rich scene representation (Hollingworth and Henderson, 2002; Hollingworth, 2007).

It seems implausible, however, that long-term memory is extensively involved in constructing goal-driven, momentary representations of scenes. Encoding into VLTM requires consolidation, the fastest type of which is “synaptic consolidation.” As it encompasses protein synthesis, synaptic consolidation takes several minutes (Dudai, 2004) – incompatible with the construction rate required for scene representation across eye movements. Moreover, VLTM, by definition, preserves information for a long time. Even though VLTM capacity may be huge, the myriad scene fragments it would have to store will unduly clutter it up. A more parsimonious solution is called for; one that combines high encoding speeds and ease of availability with a sufficiently large capacity.

To satisfy these seemingly contradictory requirements, a “proto-LTM,” or “medium-term” memory was postulated by Melcher (2006). Proto-LTM allows large amounts of information to be kept available over a period of minutes, but this information is not consolidated. At the time, no neural mechanism for such type of memory could be offered. Recent discoveries, however, suggest the existence of a retention mechanism that could satisfy these requirements. Information acquired at fixation may be retained afterward in an “activity-silent” neural state, which results in latent mental representations (Stokes, 2015; Postle, 2016).

We propose that building up a rich representation of scenes involves integration of local representations that exist in *latent memory states*. Latent representations are residual traces of former active representations. Unlike VSTM representations, latent representations do not require persistent activity for their maintenance and consequently can be much more numerous than typical VSTM capacity allows. They can quickly be retrieved by dynamical attentional prioritization, flexibly depend on task demands, and thus offer the resources necessary for obtaining a detailed representation of entire scene.

## Activity-Based and Activity-Silent Working Memory

Maintenance in working memory has traditionally been associated with increased and unremitting levels of neural activity, such as sustained neuronal spiking or persistent neural population oscillations (Brunel, 2003). This conception stems from experiments, in which animals or humans typically have to remember several items and, after a couple of seconds’ delay, memory for these items is tested. While information is retained in memory during the delay period, brain activity is found to be enhanced, in proportion to the amount of retained information (“memory load”). Results like this are taken as evidence that the memory system has to work harder to maintain more information (Postle, 2016).

However in the last 3–5 years, evidence increasingly suggests that working memory can also be maintained without elevated brain activity. An “activity-silent” neural state allows maintenance of memory representations in a hidden or latent form (Stokes, 2015). Although silent, the neural mechanism underlying such neural states is anything but inert. According to the recent understanding of synaptic connections, short-term changes in synaptic efficacy may carry memory information in absence of persistent neuronal spiking or oscillatory activity (Mongillo et al., 2008). These synaptic changes give rise to evanescent circuits that are constantly being replicated in different network locations (Routtenberg, 2013). This mechanism allows for configuration and integration of representational networks (Schacter and Addis, 2007). Accordingly, the latent representations may be maintained in the patterns of synaptic connectivity, which are based on short-term modulation of synaptic weights during encoding (Stokes, 2015; Postle, 2016).

Activity-silent and activity-based maintenance are not mutually exclusive. In fact these mechanisms may work in tandem (Stokes, 2015). In active memory maintenance, oscillatory dynamics and spiking fluctuate, disappearing and reappearing intermittently (van Leeuwen and Raffone, 2001). Between the active states, information is maintained by temporary changes in synaptic weights of the recurrent connections (Lundqvist et al., 2016; Trübutschek et al., 2017). In the time course of a typical memory task, the activity-based states are more prominent in the initial period after presentation of to-be-memorized items, which mostly involves encoding. This period is followed by activity-silent maintenance, during which, however, activity may intermittently resurface (Trübutschek et al., 2017).

The alternation of activity-based and activity-silent neural states may be controlled by attention. Attention dynamically *prioritizes* representations in working memory whenever they become relevant to behavior (Rose et al., 2016; Myers et al., 2017; van Ede et al., 2017). As a result, representations are moved from an activity-silent state into the focus of attention, i.e., into an active neural state and vice versa. Dynamical prioritization of one item does not impair the maintenance of unprioritized items, allowing the operation to be reversed (van Ede et al., 2017).

Dynamical prioritization may involve two component processes: first, selecting a memory and, second, reconfiguring its state according to the current task demands (Myers et al., 2017). Thus, maintenance of currently attended items is accompanied by enhanced activity, whereas unattended items are maintained in an activity-silent state. As soon as attention is shifted to them, they switch to an active state. This allows latent representations to be temporally precise, i.e., items appear in the focus of attention at the most relevant times (van Ede et al., 2017).

## Latent Memory Representations are Building Blocks of Scene Representations

Rapid shifts of attention are a key feature of eye movement control. Just before the onset of a saccade, attention shifts focus to the new fixation target (Deubel and Schneider, 1996). Visual information about the new fixation target is acquired after the saccade, as long as the target remains in focus. The new information is encoded in working memory by active neuronal firing, which results in temporary change of synaptic weights (Nee and Jonides, 2013; Trübutschek et al., 2017). Toward the end of the fixation, transfer of attention to the next fixation location deprioritizes the current item. Subsequently, its representation turns from an active to an activity-silent, latent state, while firing activity encodes the information at the new fixation location.

Items that are preserved in a latent state have elsewhere become known as “accessory memory items” (Olivers et al., 2011). Unlike in scene perception, however, these were not supposed to be kept for immediate use. This, however, is the essential role of our latent representations. Our key hypothesis is that building up a scene representation involves integration of multiple latent memory representations retained across eye movements, in order to support fine-grained operations with task-relevant items of the scene. Thus, latent representations carry information about recently visited items that are directly related to completion of the visual task at hand.

The latent representation does not require sustained activity for maintenance and is, therefore, energy-efficient. This enables significantly larger number of items to be retained than in a classical VSTM. But this number is still limited to task-relevant ones, as attention operates as a gatekeeper; representations can only enter the latent state from a prior activated state. This keeps scene representation relatively sparse and selective, opening the door for effects of attentional and change blindness (Rensink, 2000).

The selectivity of a latent representation is a function of the distribution of attention within the entire visual field. The spatial extent of the attention field scales with eccentricity (Puckett and DeYoe, 2015) and its size and position is flexible depending on the current visual task (Herrmann et al., 2010). It will allow, for instance, more than one location to be selected at once. In visually demanding tasks, there is a tradeoff between the number of items in memory and the precision of their selection: the more precision is required, the fewer locations can be selected at once (Hogeboom and van Leeuwen, 1997; Franconeri et al., 2007). If a task requires high precision, the latent representation includes more details about the items represented, at the expense of their quantity. Vice versa, when precision could be sacrificed, more than a few locations could be selected at once.

The selectivity of latent representation renders it unlikely to contain many low-level sensory characteristics. However, it likely preserves the heterogeneity of the visual field, which may be shaped by the typical eccentricity-dependent degradation of acuity and color sensitivity from fovea to periphery. Furthermore, memory capacity is much larger for scene layout information than for single objects in a scene (Sanocki et al., 2010). Therefore, latent representations may come associated with a substantial amount of scene layout information.

A series of latent representations should remain in memory long enough to enable task completion, e.g., for several tens of eye movements. Because multiple latent representations coexist in time, this allows them to interact. Such interactions may have automatic and implicit effects on the performance of the current task. Moreover, the interaction may result in a characteristic form of location priming, which may be called *latent priming*. Since the latent representations are accumulated across sequential eye movements, the locations encoded at the previous representation may prime that for the following one. This is in analogy to priming as known in scene perception. A scene presented as a prime facilitates subsequent spatial processing of target objects within a following test scene. This finding was explained by activation of a prime-induced representation of the scene’s layout, a representation which integrates information about task-relevant objects and their layout (Sanocki, 2003).

Since latent memory representation is based on changes of synaptic weights, these may be the loci of integration of sequential views. During a sequence of fixations, the weights of synaptic connections established at prior fixations will be modulated by subsequent ones. The strength of modulation may depend on the behavioral relevance of the fixated item, as expressed by the amount of attention it received. The integration may involve changes in supple synapses, i.e., synapses that are highly malleable and give rise to evanescent circuits (Routtenberg, 2013). These transient memory networks do not depend on consolidation and so there is no need to retrieve them – this provides instant access and high flexibility to meet the momentary visual goals.

Our hypothesis is consistent with an understanding of working memory without separation between processing and information storage units. Previous information continually modulates the presently processed one. This results in the active and ongoing accumulation and integration of information that occurs in natural stimulation conditions (van Leeuwen and Raffone, 2001; Hasson et al., 2015; Voss et al., 2017).

Whereas latent representations support completion of the task at hand, after many repetitions of a task they may enter long-term memory storage. There, latent representations may be retained as experience instrumental for handling a category of similar tasks. This experience may constitute the “selection history” that biases attentional prioritization of items previously attended in similar contexts (Awh et al., 2012). The “selection history” may contradict current selection goals. As a result, complex interactions may occur between “selection history” and latent representations related to the current task.

## Detecting Scene Buildup Across Eye Movements

Studying the integration of scene information across saccades has been made possible by recent advances in combining video-based eye-tracking with EEG measurement. Eye movement-EEG co-registration allows addressing research questions inaccessible with either technique alone. Visual processing can now be studied in naturalistic visual conditions – a major step from traditional stimulus-response paradigms. Consequently, co-registration is increasingly being adopted for studying, for example, memory encoding (Nikolaev et al., 2011), reading (Dimigen et al., 2011), attention (Fischer et al., 2013), visual search (Körner et al., 2014), and emotional responses (Simola et al., 2015).

Co-registration of free viewing behavior is methodologically demanding. Sequential eye movements systematically affect EEG: EEG responses to the current saccade overlap with those to the preceding and following ones, giving rise to spurious effects. Since fixation durations have a non-uniform distribution, this problem cannot be solved by averaging EEG across fixation-related epochs (Dimigen et al., 2011; Dias et al., 2013; Nikolaev et al., 2016). The established solutions to this problem involves matching of eye movement characteristics between experimental conditions (Nikolaev et al., 2016) or statistically considering eye movement effects using Generalized Additive Mixed Modelling (GAMM) (Van Humbeeck et al., 2018).

To reveal latent memory representations across eye movements, EEG-eye movement co-registration can be combined with methods allowing the detection of activity-silent neural states. We suggest two major approaches. The first is based on assumption that latent representations are maintained in patterns of functional connectivity (Stokes, 2015). Functional connectivity is manifested in frequency-specific patterns of phase synchrony, which support neural communication and plasticity (Fell and Axmacher, 2011). Accordingly, the analysis of functional connectivity during fixation intervals may reveal scene buildup from latent representations.

Synchrony measures of scalp EEG are sensitive to various stages of the activity-based memory process. Synchrony is higher during encoding for information remembered than during encoding for information subsequently forgotten (Summerfield and Mangels, 2005). During memory maintenance, widespread increase of synchrony is proportional to memory load (Payne and Kounios, 2009). Recently we explored the dynamic reconfiguration of functional connectivity in free viewing during encoding and retrieval (Seidkhani et al., 2017). We evaluated the functional connectivity after fixation onset through graph-theoretical measures. Encoding involved a more segregated mode of operation than retrieval, as it was evident from such measures as mean path length, radius, closeness, and eccentricity.

However, since between-area synchronization is a prerequisite of memory formation (Fell and Axmacher, 2011) EEG synchrony may also reflect activity-silent retention. To reveal latent representations across eye movements, functional connectivity analysis could be applied to fixation intervals in free viewing exploration of a scene, for instance preceding a memory test.

The other approach proposed for identifying latent representations involves multivariate pattern analysis (MVPA). Initially developed for and intensively used in fMRI research, MVPA has been increasing in popularity for application to EEG/MEG (King and Dehaene, 2014; Trübutschek et al., 2017; Wolff et al., 2017). Multiple data points are jointly analyzed in order to isolate the topographical patterns that differentiate best the experimental conditions. MVPA is implemented via machine learning, where a classifier is trained to *decode* specific mental states from the patterns of brain activity. To extract time-course information from EEG, a series of classifiers can be trained, each applied on successive time slices of the data allowing the researcher to trace how mental representations unfold over time.

The activity-silent representation during retention interval could be divulged by presenting a probe impulse, which pings a hidden neural state. MVPA of the EEG response to this impulse could revive item-specific activity, like observed during an item’s encoding (Stokes, 2015; Postle, 2016). For example, Wolff et al. (2017) asked participants to remember two oriented grating stimuli. Then, a cue indicated which of the stimuli will be tested after a 1 s retention interval. During the retention interval, a probe impulse was flashed. The EEG response to this impulse, decoded with MVPA, reflected not only the attended (cued) but also the unattended (uncued) stimulus, which resided in a hidden state of memory. An MVPA experiment for detection of latent representations across eye movements may involve gaze-contingent presentation of probe (flash) impulses during scene inspection.

## Conclusion

Taking up the visual information from a scene involves more than a snapshot. Visual system, memory and attention work together to achieve a goal-oriented representation. How this is achieved has been studied for decades, but has still largely remained an unresolved problem. Recent advances in the understanding of memory mechanisms, along with developments in the methodology of simultaneous recording eye movement and brain activity and novel, computationally intensive approaches to decoding hidden patterns of brain activity, offer a perspective for solving this intriguing puzzle.

## Author Contributions

AN contributed the central ideas to this paper. AN and CvL wrote the manuscript.

## Conflict of Interest Statement

The authors declare that the research was conducted in the absence of any commercial or financial relationships that could be construed as a potential conflict of interest.
